# Serum Autofluorescence, a Potential Serum Marker for the Diagnosis of Liver Fibrosis in Rats

**DOI:** 10.3390/ijms130912130

**Published:** 2012-09-24

**Authors:** Yu-Tao Zhan, Li Li, Jing Weng, Xin Song, Shao-Qi Yang, Wei An

**Affiliations:** 1 Department of Gastroenterology and Hepatology, Beijing Tongren Hospital, Capital Medical University, Beijing 100730, China; E-Mail: dadi-121@163.com; 2 Reproductive Medicine Center, Capital Medical University, Beijing 100069, China; E-Mail: weng.jing@163.com; 3 Department of Clinic Laboratory, Beijing Tongren Hospital, Capital Medical University, Beijing 100730, China; E-Mail: songxin_1103@163.com; 4 Department of Gastroenterology, the Affiliated Hospital of Ningxia Medical University, Yinchuan 750000, China; E-Mail: shaoqiynh@hotmail.com; 5 Department of Cell Biology, Municipal Laboratory for Liver Protection and Regulation of Regeneration, Capital Medical University, Beijing 100069, China

**Keywords:** serum autofluorescence, hepatic fibrosis, diagnosis, noninvasive

## Abstract

Fluctuations in serum autofluorescence (AF) intensity have recently been widely used as markers of certain diseases such as cancer. To determine the diagnostic value of serum AF intensity for liver fibrosis in rats, we induced liver fibrosis by subcutaneous injection of carbon tetrachloride into rats. The rat serum AF intensities were detected at the excitation wavelength of 337 nm and the emission wavelength of 512 nm. The degree of liver fibrosis was evaluated by Van Gieson’s staining. The relationship between serum AF intensity and the degree of liver fibrosis was analyzed by Spearman and Pearson Correlation. The diagnostic sensitivity and specificity of the serum AF was determined by analyzing the receiver operating characteristic (ROC) curves. Our results show that the serum AF intensity in the rat liver fibrosis model increased when compared with control rats eight weeks and twelve weeks post induction of liver fibrosis. However, there was no significant difference in serum AF intensity between fibrotic and control rats at four week post induction. Furthermore, serum AF intensity correlated positively with the severity of the degree of hepatic fibrosis. ROC analysis further suggested that serum AF intensity is a valid marker for staging fibrosis. Therefore, it may potentially be developed as a novel diagnostic tool for hepatic fibrosis.

## 1. Introduction

Hepatic fibrosis may progress to liver cirrhosis [1], which is a severe disease leading to death. If patients with hepatic fibrosis are subjected to early treatment efficiently, hepatic fibrosis can be reversed [2]. Liver cirrhosis, however, is generally irreversible. Therefore, the diagnosis of hepatic fibrosis at early stage and the subsequent efficient treatments are very important for the prevention of cirrhosis. Hepatic fibrosis may be caused by various chronic liver diseases, including chronic hepatitis B or C, alcoholic liver disease, and nonalcoholic steatohepatitis, *etc.* The diagnosis and evaluation of hepatic fibrosis is thus of great clinical value. The diagnostic methods for hepatic fibrosis include invasive and noninvasive methods. The invasive method refers mainly to liver biopsy (LB). Up to now, LB has been considered as a unique and reliable tool for the diagnosis of hepatic fibrosis and the gold standard method of staging fibrosis [3]. LB, however, is an invasive and expensive procedure that often is not readily accepted by patients [4], particularly by patients to which repeated LB are performed to evaluate anitfibrotic therapy. Moreover, due to the high intra-observer variation among pathologists for the staging of liver biopsy specimens, it is debatable whether LB can be used for the accurate assessment of hepatic fibrosis [5–8]. In addition, LB can cause potential complications, such as bleeding in the liver and around the site of the procedure, pain around the biopsy area, infection, and damage to liver tissue, *etc*. There are two main kinds of noninvasive methodologies for the evaluation of hepatic fibrosis. One is using serum markers, including indirect and direct markers; and the other one is the application of various imaging techniques. Indirect serum markers are based on single or algorhythmic elaboration of commonly observed alterations in liver function that do not necessarily reflect extracellular matrix metabolism. Direct serum markers reflect actual extracellular matrix turnover within the liver. Although the serum marker test is noninvasive, it fails to diagnose non-significant hepatic fibrosis accurately and its clinical diagnostic value remains controversial. Imaging techniques that can be used for the diagnosis of hepatic fibrosis include ultrasound, computed tomography (CT) scan, magnetic resonance image (MRI), and transient elastography. Currently, CT and MRI can indicate the presence of cirrhosis with high specificity but with very low sensitivity, and they do not allow any delineation of the fibrotic stage. Transient elastography can only diagnose liver cirrhosis and severe fibrosis, and its diagnostic accuracy for early stages of fibrosis is poor. In conclusion, no ideal diagnostic methods for hepatic fibrosis have been developed. As a result, sensitive and specific tools for the noninvasive evaluation of hepatic fibrosis remain a high requirement.

In recent years, several studies showed that AF intensity is elevated in patients with tumors [9,10]. Using the ratio of red to green autofluorescence, the algorithm identified tissues clinically determined to be cancer or tissues clinically suspicious for neoplasia with a sensitivity of 90% and a specificity of 87% [11]. However, little is known about serum AF intensity in patients or animals with hepatic fibrosis. In preliminary experiment, we found that AF intensities at excitation/emission wavelength of 337 nm/512 nm, 337 nm/450 nm, and 370 nm/440 nm are elevated in patients with cirrhosis, especially at 337 nm/512 nm. In this study, we induced hepatic fibrosis in rats and analyzed the relationship between the alternations in AF intensity and the severity of hepatic fibrosis. We found that serum AF intensity at the excitation/emission wavelength of 337 nm/512 nm is highly correlated with the degrees of hepatic fibrosis. The sensitivity and specificity of serum AF intensity for diagnosing hepatic fibrosis increase with the progressing of hepatic fibrosis. The serum AF test, therefore, may become a potential diagnostic method for hepatic fibrosis.

## 2. Results

### 2.1. Serum AF Intensity

Although the serum AF intensity in the fibrosis model group rats also increased compared with that in the control group rats at the 4th week, no significant difference between the two groups was found (*p* > 0.05, Table 1). However, the serum AF intensities in rats with fibrosis increased significantly at the 8th and 12th week (*p* < 0.01).

### 2.2. Pathological Changes of Liver Tissue

**Figure 1:**
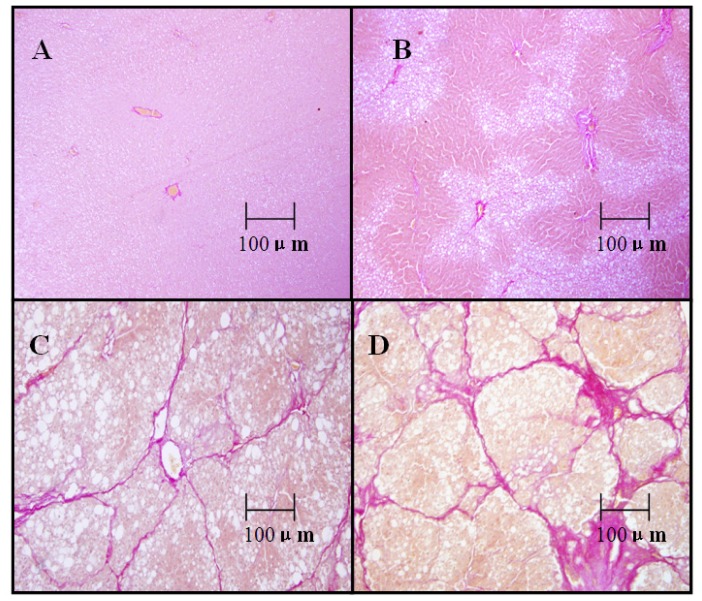
Pathological changes in liver at different fibrotic stages. (**A**) Control group rats, thin fibers were seen around blood vessels or bile ducts; (**B**) fibrotic model group rats at the 4th week, thin collagen fibers extended into the fatty degeneration area; (**C**) model group rats at the 8th week, the hepatic lobule is enveloped incompletely by thin collagen fibers; (**D**) model group rats at the 12th week, large amount of fibrous connective tissue was observed, forming typical pseudolobules.

Van Gieson’s staining clearly showed that little collagen was distributed around the blood vessel wall or bile duct wall in livers of control rats (Figure 1A). Thin collagen fibers were observed in liver rats with fibrosis starting from the 4th week (Figure 1B). At the 8th week, the hepatic lobule was incompletely enveloped by thin collagen fibers (Figure 1C). The liver damage progressed further at the 12th week, showing a larger accumulation of fibrous connective tissue and the formation of typical pseudolobules (Figure 1D).

### 2.3. Relationship between Serum AF Intensity and Degree of Hepatic Fibrosis

**Figure 2:**
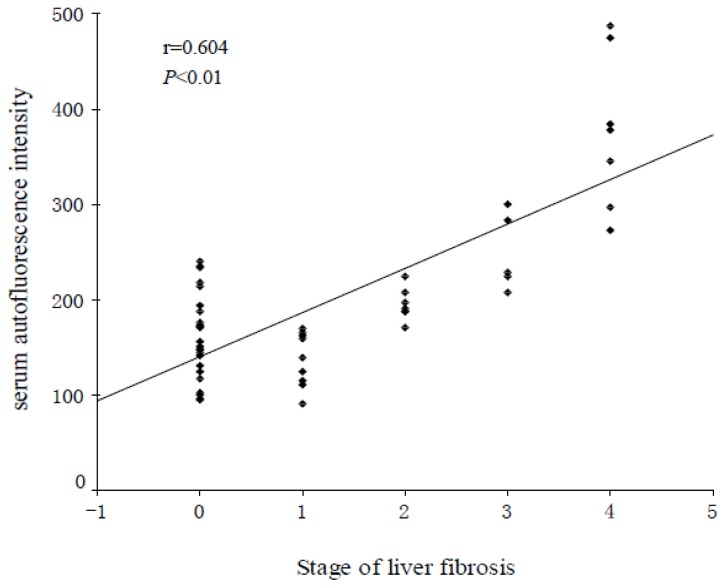
Relationship between serum autofluorescence (AF) intensity and stage of liver fibrosis. A steady gradual increase in serum AF intensity was observed with increasing severity of liver fibrosis (*p* < 0.01).

Figure 2 shows that the serum AF intensity increased gradually with the progression of hepatic fibrosis, and correlated positively with hepatic fibrosis (*r* = 0.604, *p* < 0.01).

### 2.4. The Sensitivity and Specificity of Serum AF Intensity for Diagnostic Hepatic Fibrosis

Table 2 shows that the sensitivity and specificity of serum AF intensity for diagnosing hepatic fibrosis increase with the progression of the degree of hepatic fibrosis.

## 3. Discussion

The diagnostic methods of hepatic fibrosis include LB, imaging methods, and serum marker. LB is the current gold standard for the diagnosis of hepatic fibrosis [12]. However, LB also has a few limitations. First, the biopsy procedure results in pain in 24.6% of patients and poses risk of severe complications in 0.31% of patients [13]. Second, it has been shown that there is a high inter- and intra-observer variation among pathologists in determining the stage of liver fibrosis using biopsy specimens [5]. Third, histological staging is based on a biopsy specimen that represents at most 1/50,000 of the total liver mass [14], and the distribution of fibrosis in the liver parenchyma is heterogeneous, which results in a non-negligible sampling error. Siddique *et al.*, found that different LB specimens (at least 15 mm long) taken at the same puncture site often indicate different liver fibrosis stages in 45% of studied patients [8]. Fourth, LB is not well accepted by patients. A French survey recently showed that approximately half of patients with hepatitis C virus infection refuse to be referred to hepatologists for fear of LB [4]. Imaging methods for diagnosing hepatic fibrosis include ultrasound, CT, MRI and transient elastography (FibroScan). It has been demonstrated that ultrasound, CT, and MRI are inadequate to diagnose and differentiate early stages of fibrosis, and the diagnosis of cirrhosis is often based on signs of advanced liver cirrhosis. FibroScan is a novel, noninvasive, and rapid bedside method for assessing liver fibrosis by measuring liver stiffness. Although recent data supports the diagnostic value of transient elastography for hepatic fibrosis, it can only diagnose liver cirrhosis and severe fibrosis, and the diagnostic accuracy for early stages of fibrosis (Metavir score F1 and F2) is poor [15]. In addition, FibroScan may not give accurate diagnosis for hepatic fibrosis in obese patients and cannot detect cirrhosis in patients with acute liver damage. Moreover, since liver stiffness is likely to change with age, the patient’s age affects the accuracy of FibroScan as well [16]. Serum marker of liver fibrosis is closely linked to the pathophysiological abnormality of fibrogenesis. Hepatic fibrosis is characterized by an excessive deposition of extracellular matrix (ECM) proteins caused by both increased synthesis and decreased or unbalanced degradation of ECM [17], which lead to higher levels of circulating ECM components or their fragments in the peripheral blood. Serum markers identified as having diagnostic value for hepatic fibrosis mainly include hyaluronic acid (HA), laminin, fibronect, procollagens I and III, and type IV collagens. Among these markers, HA has been considered to have good accuracy for the diagnosis of hepatic fibrosis. Other serum markers related to the mechanism of hepatic fibrosis such as matrix metalloproteinases (MMP), tissue inhibitors of metalloproteinases (TIM), transforming growth factor β (TGF), and tumor necrosis factor (TNF) β may have low sensitivity and specificity. The ideal marker should have the following characteristics: (1) specific and sensitive for liver fibrosis; (2) accurate staging of hepatic fibrosis; (3) unaffected by comorbidities; (4) reproducible; (5) non-invasive; and (6) cost effective. So far, serum markers that possess all the advantages described above for the diagnosis of hepatic fibrosis have not been identified.

Many serum proteins and non-protein molecules without exogenous fluorescent substances can produce AF after UV (ultra-violet) excitation at the appropriate wavelength. AF detection has three major advantages over other light-based investigation methods: highly sensitive, quick, and safe. AF detection has been used recently in the field of cancer diagnosis. Masilamani *et al.*, [18] found that blood components of patients with gastric cancer, breast cancer, and Hodgkin’s lymphoma showed distinct and enhanced fluorescence intensity (around 630 nm) due to the porphyrin fluorophore. However, serum AF has never been indicated to be applicable in hepatic fibrosis diagnosis. Here we demonstrate significantly higher serum AF intensity in liver fibrotic rats compared with normal rats, and there is a significant positive correlation between serum AF intensity and hepatic fibrosis stages. Many experts consider non-invasive tests for fibrosis with an AUROC value of 0.85–0.90 to be as good as liver biopsies for staging fibrosis [19]. The AUROC values for serum AF tests for *F* ≥ 2 (0.876), *F* ≥ 3 (0.994), and *F* = 4 (0.997) are higher than 0.85, suggesting that serum AF is a good marker for staging fibrosis.

Serum AF detection for diagnosis of hepatic fibrosis has a number of advantages. First of all, it is non-invasive when compared to LB. Secondly, it is simple, rapid, and economical when compared to traditional serum markers, such as HA detection. For HA detection, Antibody kits, and antigen-antibody reaction time are required. Therefore, serum AF detection may potentially become a better diagnostic method for hepatic fibrosis, especially severe hepatic fibrosis.

So far it is unclear what substance in hepatic fibrosis rat serum generates autofluorescence at 512 nm when excited at 377 nm. In this study, rat liver fibrosis was induced by carbon tetrachloride that can induce lipid peroxidation products. Lipid peroxidation products play an important role in the pathogenesis of liver fibrosis. Lipid peroxidation products have a maximum excitation wavelength at 360 nm and a maximum emission wavelength at 440 nm [20]. Advanced glycation end products have the characteristics of autofluorescence, and they increase in serum of patients with cirrhosis, but they generate more intense fluorescence at 440 nm when excited at 370 nm [21,22]. The excitation peak and emission peak of serum bilirubin are 460 nm and 515 nm, respectively. Collagen type I and type V have AF of emission maxima at 430 and 480 nm after excitation at 332 nm [23]. All data above suggest that the substance producing autofluorescence (excitation wavelength 337 nm, emission wavelength 512 nm) may not be lipid peroxidation products, advanced glycation end products, bilirubin, or collagen type I and type V. It is speculated that other ECM components may be the substance of serum autofluorescence in rats with liver fibrosis. Further investigation is needed to elucidate the source of serum autofluoresecnce.

## 4. Experimental Section

### 4.1. Animals and Sample Preparation

Sprague-Dawley (SD) rats (weight between 180 g to 220 g) obtained from Experimental Research Unit, Capital Medical University Animals used in the experiments were treated with humanity and experimental protocols were approved by Human and Animal Ethics Committee, Capital Medical University. Fifty-four male adult SD rats were randomly divided into two groups: control group and hepatic fibrosis group. The experimental procedure was conducted with the approval of the Ethics Committee of Capital Medical University. CCl_4_ was purchased from Beijing Chemical Reagents Company and prepared by dilution with pure olive oil (ratio 4:6). All rats were allowed to acclimatize for a week before experimentation. Thirty rats were subcutaneously injected with CCl_4_ (0.3 mL/kg body weight) every other day throughout the entire period of the experiment. The control group received subcutaneous pure olive oil alone. At the 4th, 8th and 12th week, ten rats in the hepatic fibrosis group and eight rats in the control group were randomly sacrificed by intraperitoneal injection of chloral hydrate (100 mg/kg). Blood samples were collected before the removal of the rats’ livers and centrifuged at 3000*g* per min for 15 min at 4 °C. The resulting serum was kept at −20 °C for serum AF intensity tests. Liver tissues were fixed in 10% formalin, embedded in paraffin, sectioned and routinely stained with Hematoxylin-Eosin.

### 4.2. Assessment of Serum AF

Serum (0.5 mL) was diluted with H_2_O (2.5 mL). After mixing, an aliquot of 3.0 mL serum sample was transferred into quartz cuvette, and analyzed using a spectrofluorometer (RF 5000, Shimadzu Corporation, Kyoto, Japan). An excitation wavelength of 337 nm was used for all samples and serum AF intensity was detected at 512 nm. All the procedures were performed at room temperature and all experiments were performed independently three times.

### 4.3. Liver Histology

To evaluate the degree of hepatic fibrosis, the liver sample was stained using the Van Gieson’s staining method. The fibrosis score was independently accessed by two pathologists who were blinded from the experiment protocol. The degree of hepatic fibrosis was semi-quantitatively assessed according to the METAVIR scoring system [24,25]. Fibrosis was staged on a 0–4 scale as following: F0 = no fibrosis; F1 = portal fibrosis without septa; F2 = portal fibrosis and few septa; F3 = numerous septa without cirrhosis; F4 = cirrhosis.

### 4.4. Statistical Analysis

For the comparison of the two groups, continuous variables were analyzed by Student’s *t* test. The relationship between serum AF intensity and the degree of hepatic fibrosis was analyzed by Spearman and Pearson Correlation. The diagnostic sensitivity and specificity of serum AF was assessed by analysis of receiver operating characteristic (ROC) curves. The most commonly used index of accuracy is the area under the ROC curve (AUROC), in which values close to 1.0 indicate high diagnostic accuracy. A two-sided *p* value < 0.05 was considered statistically significant. All statistical analyses were carried out using the statistical software package SPSS, version 12.0 (SPSS, Inc., Chicago, IL, USA, 2003).

## 5. Conclusions

This study showed that serum AF intensity (excitation wavelength 337 nm, emission wavelength 512 nm) correlated positively with the degree of severity of hepatic fibrosis, and is sensitive and specific to moderate or severe hepatic fibrosis (*F* ≥ 2). The serum AF test is noninvasive, simple, rapid and economical. Serum AF, therefore, would be clinically useful as a non-invasive marker of hepatic fibrosis. The substance that generates serum autofluorescence (excitation wavelength 337 nm, emission wavelength 512 nm) in hepatic fibrosis rats needs to be identified.

## Figures and Tables

**Table 1:** Serum autofluorescence (AF) intensity in rats with normal liver and liver fibrosis group.

Groups	4th week	8th week	12th week
Control	115.57 ± 21.01	159.23 ± 19.52	207.85 ± 31.06
Fibrosis	146.05 ± 38.26	207.39 ± 28.88 △	368.68 ± 78.84 △

Values are expressed as mean ± SD.

△ *p* = 0.001 (8th week); 0.000 (12th week), *p* < 0.01, compared with control group.

**Table 2:** The diagnostic sensitivity and specificity of serum AF intensity for determining hepatic fibrosis degree.

Fibrosis degrees	AF intensity
*F* ≥ 1(F0 *vs.* F1-2-3-4)	

AUROC	0.710 (0.569–0.851)
CUT-OFF VALUE	81.869
sensitivity	0.786
specificity	0.583

*F* ≥ 2(F0-1 *vs.* F2-3-4)	

AUROC	0.876 (0.783–0.969)
CUT-OFF VALUE	92.792
sensitivity	0.889
specificity	0.765

*F* ≥ 3(F0-1-2 *vs.* F3-4)	

AUROC	0.994(0.979–1.009)
CUT-OFF VALUE	106.527
sensitivity	0.917
specificity	0.975

*F* ≥ 4(F0-1-2-3 *vs. F* = 4)	

AUROC	0.997 (0.987–1.007)
CUT-OFF VALUE	136.328
sensitivity	0.857
specificity	0.978

The diagnostic sensitivity and specificity of serum AF intensity for hepatic fibrosis were 0.786 and 0.583 for *F* ≥ 1, 0.889 and 0.765 for *F* ≥ 2, 0.971 and 0.975 for *F* ≥ 3, 0.857 and 0.978 for *F* = 4, respectively.
